# An individualized Bayesian method for estimating genomic variants of hypertension

**DOI:** 10.1186/s12864-023-09757-9

**Published:** 2023-11-07

**Authors:** Md Asad Rahman, Chunhui Cai, Na Bo, Dennis M. McNamara, Ying Ding, Gregory F. Cooper, Xinghua Lu, Jinling Liu

**Affiliations:** 1 Department of Engineering Management and Systems Engineering, Missouri University of Science and Technology, Rolla, MO USA; 2 Department of Biomedical Informatics, University of Pittsburgh, Pittsburgh, PA USA; 3 Department of Biostatistics, University of Pittsburgh, Pittsburgh, PA USA; 4 Department of Medicine, University of Pittsburgh, Pittsburgh, PA USA; 5 Department of Biological Sciences, Missouri University of Science and Technology, Rolla, MO USA; 6 Present Address: Department of Epidemiology, College of Public Health and Health Professions and College of Medicine, University of Florida, Gainesville, FL USA

**Keywords:** Individualized Bayesian inference, Genome-wide association studies, Genomic variants, Single nucleotide polymorphism, Hypertension, Blood pressure, Precision medicine

## Abstract

**Background:**

Genomic variants of the disease are often discovered nowadays through population-based genome-wide association studies (GWAS). Identifying genomic variations potentially underlying a phenotype, such as hypertension, in an individual is important for designing personalized treatment; however, population-level models, such as GWAS, may not capture all the important, individualized factors well. In addition, GWAS typically requires a large sample size to detect the association of low-frequency genomic variants with sufficient power. Here, we report an individualized Bayesian inference (IBI) algorithm for estimating the genomic variants that influence complex traits, such as hypertension, at the level of an individual (e.g., a patient). By modeling at the level of the individual, IBI seeks to find genomic variants observed in the individual’s genome that provide a strong explanation of the phenotype observed in this individual.

**Results:**

We applied the IBI algorithm to the data from the Framingham Heart Study to explore the genomic influences of hypertension. Among the top-ranking variants identified by IBI and GWAS, there is a significant number of shared variants (intersection); the unique variants identified only by IBI tend to have relatively lower minor allele frequency than those identified by GWAS. In addition, IBI discovered more individualized and diverse variants that explain hypertension patients better than GWAS. Furthermore, IBI found several well-known low-frequency variants as well as genes related to blood pressure that GWAS missed in the same cohort. Finally, IBI identified top-ranked variants that predicted hypertension better than GWAS, according to the area under the ROC curve.

**Conclusions:**

The results support IBI as a promising approach for complementing GWAS, especially in detecting low-frequency genomic variants as well as learning personalized genomic variants of clinical traits and disease, such as the complex trait of hypertension, to help advance precision medicine.

**Supplementary Information:**

The online version contains supplementary material available at 10.1186/s12864-023-09757-9.

## Background

Hypertension (HTN; high blood pressure) is a key risk factor for many cardiovascular diseases, and it was primarily responsible for about 7.8 million world-wide deaths in 2015 alone. Previous studies indicate that in addition to environmental factors, genomic factors play a significant role in blood pressure (BP) regulation [1]. Hypertension is a polygenic disease [2], burdening a large population across the globe. Current efforts at identifying significant genomic variants mostly involve genome-wide association studies (GWAS). Although GWAS has successfully identified more than 1000 genomic loci containing significant single nucleotide polymorphisms (SNPs; the most common type of genomic variants among people) for BP regulation [3], there are limitations to this commonly used approach. GWAS requires a large cohort to gain enough power to identify many of the significant SNPs, especially those with low minor allele frequency (MAF). That is why before 2015 only about 64 significant SNPs were identified for blood pressure, and only recently were more SNPs identified due to the increased sample sizes (~ 1 million individuals) [4–6]. Still, most SNPs identified so far are common SNPs with small effect sizes, and the total genetic variance in blood pressure explained by these ~1000 SNPs is small (~5.7%) [3]. There are likely a significant number of non-common variants missed by GWAS that can help explain much of the remaining genomic variance [7]. 

GWAS is a population-based approach, and it extracts significant SNPs from a population level, not considering the specific genome of a given individual. Therefore, GWAS is not tailored to identify the genomic influences of HTN in an individual, which is the focus of personalized medicine. It is not uncommon that a HTN patient does not carry the disease-associated alleles (mostly minor alleles) of any significant variants identified at the population level. Thus, identifying the most probable genomic variants of individual patients is important but remains an unmet need.

We have developed an individualized Bayesian inference (IBI) algorithm for estimating the genomic factors influencing the development of hypertension and other complex traits in an individual. As a general machine learning framework, IBI applies a Bayesian method to identify the significant genomic variants in a given individual or patient. Bayesian methods including Bayesian multiple logistic [8] or linear regression have been used for identifying the causal SNPs among the significant genomic regions identified by GWAS for binary or continuous traits [9, 10]. However, none of these are individualized. IBI evolved from a tumor-specific causal inference algorithm (TCI) that members of our team developed for estimating the somatic mutations driving the development of individual cancerous tumors [11]. In contrast to TCI, IBI is designed to model and learn the relationships between an individual genome and a complex trait, such as HTN. Also, IBI was optimized for efficient computation with whole-genome data, whereas TCI was developed to use whole-exome data.

IBI identifies significant and potentially causal genomic variants for each individual based on their specific genomic background (and available training data on similar individuals). By concentrating on the genomic variants observed in a particular individual, IBI has the potential to discover significant variants of low frequency that exist only in a small number of individuals and could have been missed by GWAS. The genomic variants identified as being significant by IBI could help inform the design of personalized treatment for individuals with or at risk for hypertension.

## Methods

### Overview of Bayesian networks

A Bayesian network (BN) [12, 13] is a probabilistic graphical model with two components. One is a graphical structure containing nodes and directed edges. Nodes represent domain variables such as genomic variants or clinical traits. Directed edges represent conditional dependencies between variables. The other component of a BN is a set of parameters which are conditional probabilities. Each node has a conditional probability given its potential causes, which can be described by a conditional probability function. The joint probabilities of all nodes can be written as a product of each node’s conditional probability, given its direct causes, based on the local causal Markov condition. A BN is a flexible framework for modeling the probabilistic relationships among variables in a complex domain by representing the joint probability of all the variables modeled in a probabilistic structure. A bipartite BN is a particular class of BN with less complexity. There are only two sets of nodes in level 1 and level 2, and potential causal relationships only occur from nodes in level 1 to nodes in level 2.

How do we search for the most probable BN given data? A very popular class of methods are score-based algorithms that assign a Bayesian score to the BN model and return the BN with the highest score [12, 13]. This Bayesian score of the BN model is assigned based on how well this BN is supported by both the data and prior knowledge [14]. In this study, we use a popular Bayesian score for modeling discrete variables, the Bayesian Dirichlet equivalent uniform (BDeu) score [14] as TCI did [11].

### The general framework of individualized Bayesian inference.

**Fig. 1:**
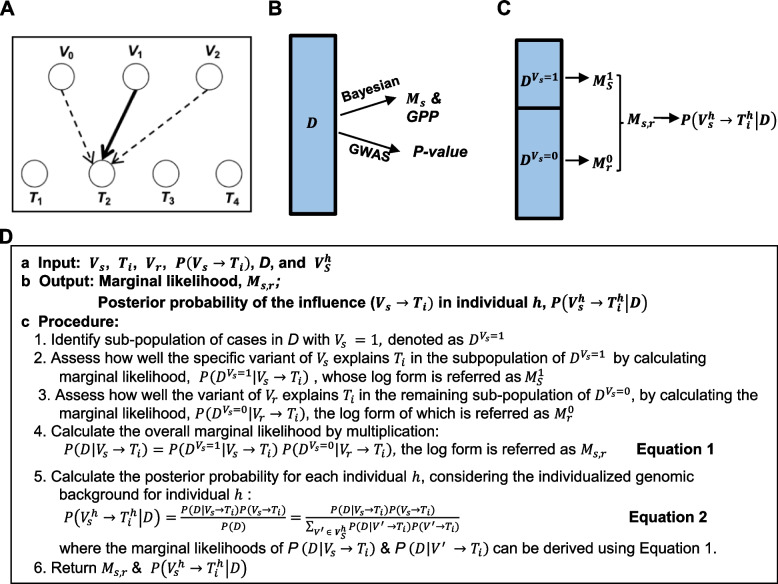
The Individualized Bayesian Inference (IBI) algorithm. **A** IBI uses a bipartite BN to model the probabilistic relationships from genomic Variants to Traits. *V* nodes denote variants and *T* nodes denote traits; the arcs denote probabilistic influence of *V* on *T* with only one assumed influencing variant being evaluated at a time indicated by the solid arc. **B** Using the entire dataset (*D*) (e.g., the population) to evaluate the association of a particular genomic variant and a trait, GWAS methods output a *p*-value while the Bayesian method uses the marginal likelihood (${M}_{s}$) and global posterior probability (GPP). **C** Based on the value of a particular variant ${V}_{s}$, IBI partitions the whole population into two subpopulations, ${D}^{{V}_{s}=1}$ and ${D}^{{V}_{s}=0}$, and derives the subpopulation-specific marginals, ${M}_{S}^{1}$ and ${M}_{r}^{0}$*,* using ${V}_{s}$ and ${V}_{r}$. The overall marginal ${M}_{s,r}$ and the individual-specific posterior probability, $P\left({V}_{s}^{h}\to {T}_{i}^{h}|D\right)$ for the SNP ${V}_{s}$ can be further derived. **D** Pseudo code for the IBI algorithm

As mentioned, IBI is based on TCI [11] and has been further developed and adapted to fit the circumstances of modeling a variety of complex diseases or traits using whole-genome genotyping or sequencing data. IBI is designed to estimate the significant genomic variants, such as SNPs, in a specific individual or patient for downstream clinical and molecular phenotypes. IBI uses a bipartite BN [12, 13] for modeling the probabilistic dependency relationships between the genomic variants as a set of nodes $V$ and the downstream traits or phenotypes as a set of nodes $T$; directed edges between nodes in $V$ and $T$ represent probabilistic dependency from variants to traits (Fig. 1A). Within this bipartite BN, among all the variants in one individual, IBI assigns a posterior probability that there is a dependency relationship between each variant (represented by a $V$ node) and the phenotypic trait of interest (represented by node $T$) specific to this individual (Fig. 1A, D).

For a current individual $h$, let ${V}_{s}$ be a variable that represents a specific genomic variant *s* (e.g., a SNP) and let ${T}_{i}$ be a specific trait *i* (e.g., HTN) of this individual. Let ${{\varvec{V}}}_{S}^{h}$ be a vector representing all the genomic variants in individual $h.$ For each possible variable ${V}_{s},$ we will examine the relationship ( ${V}_{s}\to {T}_{i}),$ which models that ${V}_{s}$ has a probabilistic influence on ${T}_{i}$. That is, the value of ${V}_{s}$ influences our belief (probability) about the distribution of ${T}_{i}$. It is this sense of the term *influence* that we will use in this manuscript. Let $P\left({V}_{s}\to {T}_{i}\right)$ be the prior probability for ${V}_{s}$ influencing ${T}_{i}$*,* which could be estimated using biological background knowledge or could be set using a uniform prior that assumes all the genomic variants have the same prior probability of influencing ${T}_{i}$*.* Let *D* represent the data. Let ${M}_{s}$ represent the log form of the marginal likelihood of $P\left(D |{V}_{s}\to {T}_{i}\right)$ which is derived by modeling one genomic variant *s* as influencing ${T}_{i}$ in the population represented by *D*. ${M}_{s}$ can be normalized by dividing by the summation of ${M}_{s}$ across all the SNPs to derive a posterior probability (PP) $P\left({V}_{s}\to {T}_{i}|D\right)$. When scoring ${V}_{\mathrm{s}}\to {T}_{i}$ for the entire population *D* using Bayesian learning and a uniform prior, PP is proportional to ${M}_{s}$; thus, the ranking of the specific driver ${V}_{s}$ by ${M}_{s}$ or PP as a potential estimator of a trait at the population level is the same. Thus, we will use ${M}_{s}$ as the score for ${V}_{\mathrm{s}}\to {T}_{i}$. When evaluating the influence of ${V}_{\mathrm{s}}$ on ${T}_{i}$ in the entire population using GWAS, the *p*-value is derived to indicate the significance of that influence (Fig. 1B).

IBI partitions the overall population into two subpopulations (Fig. 1C). Suppose the current patient has ${V}_{s}=1$, which represents the minor-allele of this SNP. Let ${D}^{{V}_{s}=1}$ represent the patient-like-me subpopulation, where all the patients in this subpopulation contain the value ${V}_{s}=1$. IBI evaluates how well ${V}_{s}$ influences the HTN status within ${D}^{{V}_{s}=1}$, which has a marginal likelihood score of $P\left({D}^{{V}_{s}=1} |{ V}_{s}\to {T}_{i}\right)$ that we abbreviate as ${M}_{S}^{1}$ (Fig. 1C, D). Let ${D}^{{V}_{s}=0}$ represent the remaining cases that do not have ${V}_{s}=1$, but rather, have ${V}_{s}=0.$ To estimate the data in ${D}^{{V}_{s}=0}$, IBI finds the SNP ${V}_{r}$ (where “r” denotes the remaining cases) that maximizes the marginal likelihood of ${V}_{r}\to {T}_{i}$, namely, $P\left({D}^{{V}_{s}=0} |{ V}_{r}\to {T}_{i}\right),$ which we abbreviate as ${M}_{r}^{0}.$ The marginal likelihood for all of the data, given ${V}_{s}$ as an individualized estimator and ${V}_{r}$ as the best estimator of the remaining cases, is ${M}_{s}^{1} + {M}_{r}^{0}$, which we refer to as ${M}_{s,r}$ (Fig. 1C, D). This score of ${M}_{s,r}$ can be used to evaluate and rank the capability of ${V}_{s}$ in explaining the patients-like-me subpopulation that contain this minor allele as well as in helping reduce the noise for modeling the remaining subpopulation.

The marginal likelihood is computed using the BDeu score [14] (Fig. 1D, Equation 1; refer to the TCI paper [8]). Individualized posterior probabilities of the form ${V}_{s}\to {T}_{i}$ are further derived relative to the SNPs that are minor alleles in the genome of the current patient *h*. Thus, the posterior probability considers the specific genomic background of the given individual (Fig. 1D, Equation 2). In summary, IBI is individualized in the following ways: (1) The overall marginal likelihood for each relationship ${V}_{s}\to {T}_{i}$ (Equation 1) contains an individualized component that uses the subpopulation of “patients like me” that have the same variant (i.e., ${V}_{s}=1$). (2) Each individual has a unique set of genomic variants. Depending on the specific set of variants, the posterior probability for a given relationship ${V}_{s}\to {T}_{i}$ may be different in different individuals (Equation 2). The individualized nature of IBI makes it a potential tool for advancing precision medicine where personalized treatments are desired for individuals of varying genetic backgrounds. IBI is implemented in Python with vectorization and matrix operations for efficient computation involving millions of variants and has been tested on whole-genome sequencing data on the BioData Catalyst platform [15].

### Genome-Wide association studies

GWAS is the standard approach for identifying the significant variants associated with traits at the population level (e.g., *p*-value < 5 × 10^−8^ for genome-wide significance). Conventional GWAS uses standard logistic regression models or Fisher’s exact test for discrete traits [16]. We performed GWAS using Fisher’s exact test on the same datasets we applied IBI and compared results.

### Data and data preprocessing

We used the whole-genome genotyping data of Affymetrix HuGeneFocused50K from the Framingham Heart Study (FHS) cohort (dbGaP Study Accession: phs000007.v30.p11), which covered about 50K gene-centric and coding SNPs across the genome [17, 18]. We used the following functions from plink for further filtration and quality control, and have acquired 38,342 SNPs: --mind 0.03 --geno 0.03 --maf 0.01 –hwe 10e-6 –me 0.05 0.1 –sexCheck. We filled missing SNP values with the most frequent value for that particular SNP across the entire population. Dominant coding was then performed in plink, and thus, the final SNP values are 0 or 1 where 0 represents zero copy of the minor allele (risk allele) and 1 represents one or two copies of the minor allele. The focus of this paper is to estimate the risk (minor) allele SNPs that potentially cause hypertension (high blood pressure) rather than protecting the subject from hypertension. Therefore, we further removed the SNPs with a minor-allele risk ratio smaller than 1, resulting in a total of 19,276 SNPs of interest. We further computed linkage disequilibrium (LD) measures, d-prime values, for every pair of these SNPs within a specified genomic region using the R package SNPRelate [19]. The d-prime value close to 1 indicates a high level of LD between the two SNPs. We set a popular d-prime threshold of 0.2 for LD pruning. After LD pruning, 19,006 SNPs remained, indicating that the vast majority (98.5%) of the 19,276 SNPs are considered as independent.

Clinical phenotype data included harmonized systolic BP (SBP) and diastolic BP (DBP) data which were downloaded from PIC-SURE on the NHLBI BioData Catalyst platform [15]. SBP and DBP are specifically harmonized by the Trans-omics for Precision Medicine (TOPMed) Data Coordinating Center [20] by taking the average of two SBP or DBP measurements obtained at a single clinic visit. 10 and 5mm Hg were specifically added for SBP and DBP for individuals taking antihypertensive drugs [21]. If SBP>=140, or DBP>=90 or an individual was taking antihypertensive drugs, we considered this individual as having HTN and assigned ‘HTN = 1’; otherwise, we classified this individual as not having HTN, and we assigned ‘HTN = 0.’ After merging the SNP and BP data, we obtained 6,613 patients with 19,276 SNPs. We performed a stratified random split to produce an 80% training set (discovery set; 5,290 subjects) and a 20% test set (1,323 subjects), and we reserved this test set for the evaluation task.

## Results

To evaluate IBI in inferring significant genomic variants for HTN, we compared its performance to that of GWAS. As a proof of concept, we applied IBI and GWAS to the whole-genome data of Affymetrix HuGeneFocused50K measurements and harmonized phenome data of BP measurements from the FHS cohort [18] as described above.

### A Bayesian method for GWAS analysis

**Fig. 2:**
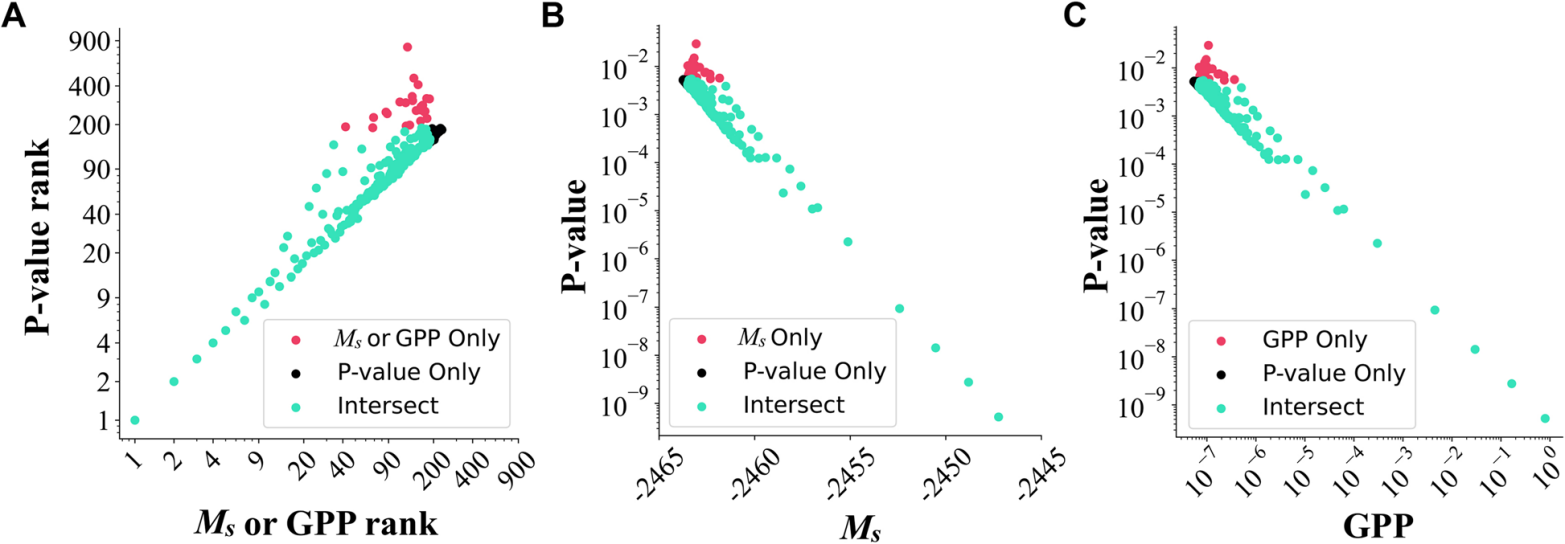
A Bayesian method for GWAS analysis. **A** The *p*-value ranks and ${M}_{s}$ or GPP ranks are the same or similar for the top 188 SNPs selected by ${M}_{s}$ or *p*-value ranking. **B** The *p*-values were negatively correlated with the ${M}_{s}$ values for the top 188 SNPs selected by ${M}_{s}$ or *p*-value ranking. **C** The *p*-values were negatively correlated with the global posterior probabilities (GPP) of the top 188 SNPs selected by ${M}_{s}$ or *p*-value ranking.

As was explained in the Methods section, when using a Bayesian method and a uniform prior to study a single variant’s influence on HTN at the population level, the derived marginal likelihood, ${M}_{s},$ for this variant is proportional to its global posterior probability (GPP), making it possible to use the marginal likelihood to find the top influencing SNP (Fig. 2A). We observed that when using a uniform prior, as we did in this study, a population-based (i.e., not individualized) Bayesian approach to identifying top-ranked SNPs based on ${M}_{s}$ yielded similar results to the population-based GWAS method (Fig. 2A). The Spearman correlation coefficient between the GWAS *p*-values and the IBI ${M}_{s}$ values across the top 188 independent SNPs (Additional File 1,2) is -0.9. We further examined and compared the top 188 independent SNPs ranked by ${M}_{s}$ or *p*-value. The top SNPs identified by high ${M}_{s}$ values or low *p*-values are highly overlapping: 164 out of the top 188 SNPs and 18 out of the top 20 SNPs overlapped between these two rankings (Fig. 2A). Furthermore, the ranking of these top SNPs by ${M}_{s}$ and *p*-value are either exactly the same or very similar (Fig. 2A) where ${M}_{s}$ and GPP are negatively correlated with the *p*-value (Fig. 2B, C).

### IBI complements GWAS and better detects significant variants of low MAF

**Fig. 3:**
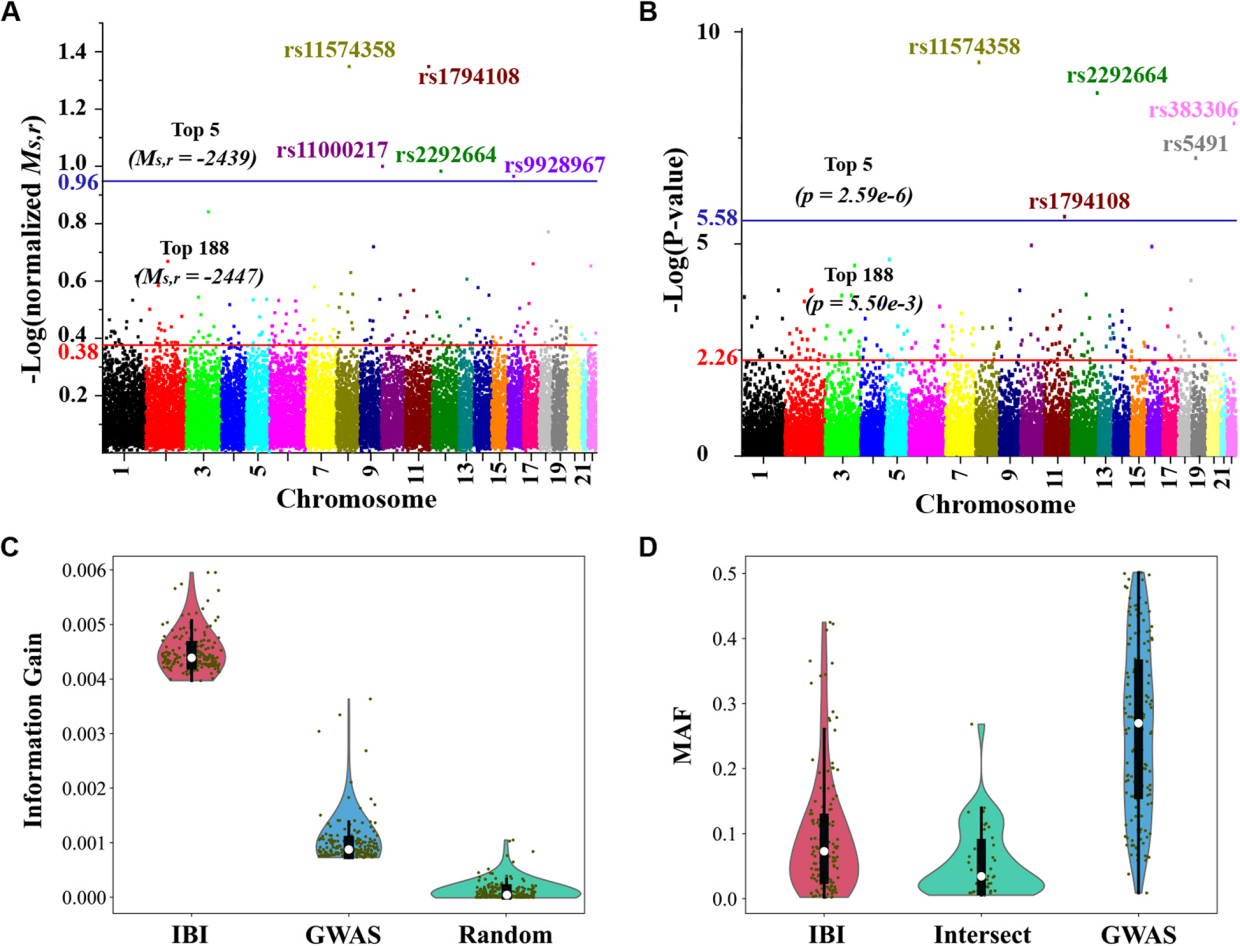
Comparison of IBI and GWAS.** A** A Manhattan plot of the chromosome location of SNPs and their normalized ${M}_{s,r}$ values according to IBI. Threshold lines for ${M}_{s,r}$ values are shown in blue and red for the top five and top 188-ranked SNPs, respectively. The top five SNPs were annotated with rs IDs. **B** A Manhattan plot of chromosome location of SNPs and *p*-values for GWAS. Threshold lines for *p*-values are shown in blue and red for the top five and top 188-ranked SNPs, respectively. The top five SNPs were annotated with rs IDs. **C** Information gain from top 188 SNPs ranked respectively by IBI, GWAS and randomly-selected 188 SNPs. The black dots represent the information gain values for individual SNPs. **D** Violin plots of the MAF distributions of the SNPs in the three groups: IBI only, Intersection, and GWAS only. The black dots represent the MAF values for individual SNPs. The thick vertical gray bars show the interquartile range and the three white dots represent the medians. Wider sections indicate higher probabilities for the given MAF values

We applied both IBI and GWAS to the training (discovery) subset of FHS subjects (*n* = 5,290) with 19,276 SNPs and HTN status, and derived the IBI marginal values of ${M}_{s,r}$ (Fig. 3A) and GWAS *p*-values (Fig. 3B) for all the SNPs in the Manhattan plots. In Fig. 3A, the values of ${M}_{s,r}$ were normalized with (-2436 - *M*_*s,r*_) / (-2436 – (-2463)) considering -2436 as the maximum and -2463 as the minimum, based on the min-max normalization technique. For the GWAS analysis, taking 0.05 / 19,276 = 2.59e-6 as the significance level for *p*-value after the Bonferroni correction, five SNPs reach such significance (Fig. 3B). In Fig. 2, the population-level ${M}_{s}$ values were derived by assuming one SNP as the global estimator or potential cause of HTN for the entire population (Fig. 1B). When using two SNPs to specifically explain HTN status from two distinct subpopulations as is done by IBI (Fig. 1C), the overall marginal values (${M}_{s,r}$) significantly increase for many SNPs of ${V}_{s}$. Among all the SNPs, 188 ${V}_{s}$ SNPs at independent loci have ${M}_{s,r}$ values bigger than the biggest ${M}_{s}$ value (indicated by the red threshold line in Fig. 3A) derived in the population level from the best global estimator, represented as ${V}_{g}$ (Fig. 3A; Additional file 3). The higher score of IBI compared to the population-based Bayesian method also has theoretical support. It has been proved that instance-based (i.e., individualized) causal inference methods, a family of algorithms to which the IBI belongs, are consistent. More specifically, in the large sample limit, the score of the data-generating instance-specific model will be assigned the highest score of any model [22]. These results support that the HTN status in the overall population has been explained better by IBI with any of the top 188 SNPs, ${V}_{s}$, explaining the subpopulation of ${D}^{{V}_{s}=1}$ and with the remaining-population estimator, ${V}_{r}$, explaining the remaining ${D}^{{V}_{s}=0}$ subpopulation, in comparison to using the best global estimator ${V}_{g}$ itself to explain the entire population of *D*.

We performed another evaluation from the perspective of information theory. In this setting, GWAS analysis is searching for a variant $${V}_{s}$$ that has strong information with respect to a trait $${T}_{i}$$(HTN), and the amount of information can be measured as information gain (IG) [23]. IG can be calculated by splitting samples according to one variable ($${V}_{s})$$ and then measuring the change in the entropy of the other variable ($${T}_{i})$$ during partitions. Rather than focusing on just one variant as in a GWAS analysis, the IBI algorithm evaluates how much information we can gain with respect to the trait $${T}_{i}$$ (HTN) if we consider both the specific variant of interest ($${V}_{s})$$ and the remaining-population estimator ($${V}_{r})$$, IG ($${V}_{s}, {V}_{r };{T}_{i}$$) [24]. The more $${V}_{s}$$ and $${V}_{r}$$ complement each other (e.g., they are two distinct estimators for different subgroups) to provide information with respect to $${T}_{i}$$, the higher the IG. In other words, IBI searches for variants that not only explain HTN well in the “patients-like-me” subpopulation, but also help enhance the information of $${V}_{r}$$ with respect to HTN in the remaining subpopulation that do not contain this specific variant of interest. Based on information gain values, the top-5 IBI SNPs were rs11574358, rs1794108, rs11000217, rs2292664 and rs9928967 while top-5 GWAS SNPs were rs11574358, rs2292664, rs383306, rs5491 and rs1794108 sharing three common ones with top-5 IBI. IG values for the top 188 IBI SNPs of $${V}_{s}$$ selected by $${M}_{s,r}$$ are significantly higher than those of the top 188 independent GWAS SNPs selected by the p-values; IG values for the top 188 GWAS SNPs are also significantly higher than the values for 188 randomly-selected SNPs as expected (Fig. 3C; Additional File 4).

We further examined whether there is any overlap between the top 188 IBI and GWAS SNPs. We found that 40 of the top 188 SNPs were identified by both IBI and GWAS, and three of the top five IBI and GWAS SNPs are the same (Fig. 3A, B). Thus, IBI and GWAS share many of the same top SNPs, suggesting a mutual agreement between these two approaches. The 148 unique SNPs for IBI or GWAS also support that the two approaches are complementary. We further examined the MAF distribution for IBI-only, GWAS-only SNPs (Fig. 3D), and SNPs common to both. Interestingly, the IBI-only SNPs overall had much lower MAF than GWAS-only SNPs (Fig. 3D). This result supports the hypothesis that IBI identifies more lower-frequency significant variants relative to GWAS by concentrating on the genomic variants of a given individual in a specific subpopulation.

### IBI discovered more individualized and diverse significant SNPs that better explain the HTN patients, compared to GWAS

For a given individual *h*, IBI derives the posterior probability for each genomic variant ${V}_{s}$, $P\left({V}_{s}^{h}\to {T}_{i}^{h }| D\right)$, by normalizing ${M}_{s,r}$ with a summation over all the ${M}_{s,r}$ across all the existing minor allele SNPs (i.e., the SNP value is 1) in this individual. This posterior probability considers the diverse genomic background or context for different individuals. More specifically, a particular SNP ${V}_{s}$ with the same ${M}_{s,r}$ may have different posterior probabilities in different individuals due to their distinct genomic background (i.e., different sets of existing minor allele SNPs). For a given HTN patient, IBI ranked all the minor alleles existing in this individual based on their individualized posterior probabilities (this ranking will be the same as the ranking based on ${M}_{s,r}$ in a given patient); the SNP with the highest posterior probability was considered to have the most probable influence on HTN for this given patient. For comparison, we designated a top SNP for each HTN patient based on the population-level *p*-values derived by GWAS: among the existing minor alleles in a given HTN patient, the non-protective minor allele with the lowest (most significant) *p*-value was considered to most probably have influence for HTN in this particular patient.

**Fig. 4:**
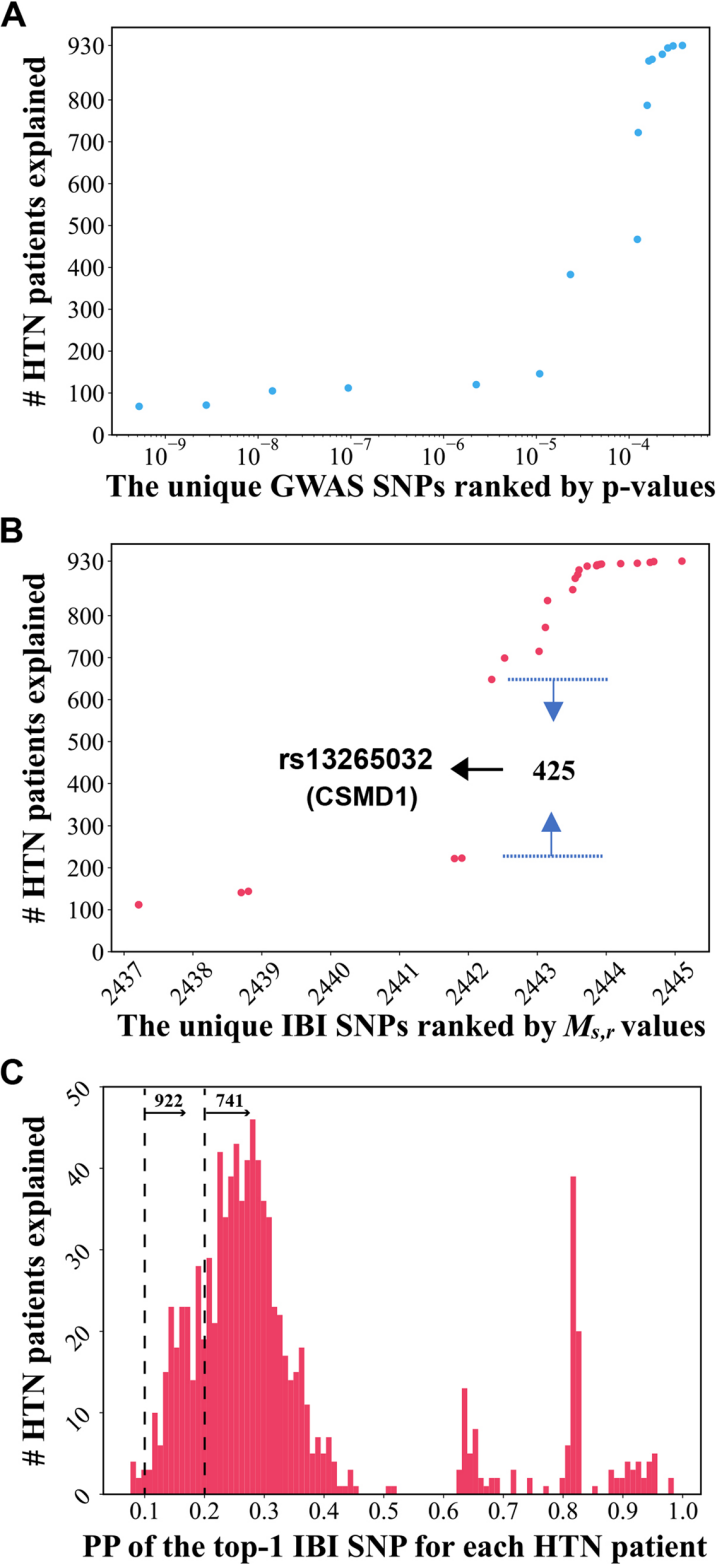
HTN patient coverage. **A** The 16 unique GWAS SNPs ranked by *p*-values are plotted against the cumulative number of HTN patients explained from the 930 HTN patients in the training (discovery) dataset. **B**. The 25 unique IBI SNPs ranked by ${M}_{s,r}$ were plotted against the cumulative number of HTN patients explained from the 930 HTN patients in the training (discovery) dataset. The number of HTN patients covered by each of these SNPs can be derived by taking the difference of the two adjacent cumulative numbers of explained HTN patients as shown for rs13265032 which accounts for 425 HTN patients. **C** A histogram of the individualized posterior probability of the top-1 SNP assigned by IBI to each of the 930 HTN patients. This shows the value scale and distribution of the individualized posterior probabilities for the top-1 SNPs across the 930 HTN patients

Among all the 930 HTN patients in the training (discovery) dataset, we identified 16 unique SNPs according to GWAS ranking (Fig. 4A; Additional File 5) and 25 unique SNPs based on IBI ranking (Fig. 4B; Additional File 6); each of these unique SNPs was assigned by GWAS or IBI as a top-1 SNP for at least one HTN patient. Figure 4A, B shows the accumulated number of explained HTN patients by including one or more of these unique SNPs; the number of HTN patients explained by each of these unique SNP can be derived by the differences of the accumulated number of explained HTN patients (Fig. 4A, B) between including and not including this particular SNP. The more unique SNPs identified by IBI (*n* = 25) suggests that IBI was able to find a more diverse set of significant SNPs with a more personalized approach. IBI identified 13 SNPs that explain less than 10 HTN patients individually while GWAS found six such SNPs. Interestingly, at the same time, IBI assigns the intronic SNP rs13265032 in the *CSMD1* loci as the top-1 SNP with the highest PP and ${M}_{s,r}$ for each of 425 (46%) HTN patients (Fig. 4B); the association of the *CSMD1* loci with HTN was further discussed later when explaining Table 1.

**Table 1:** Novel SNPs in BP-associated genes identified by IBI as the individualized and most-probable HTN cause

rs ID	Genes: Variant type	MAF	IBI rank	GWAS rank	*p*-value	# HTN explained
rs13265032	CSMD1: Intron Variant	0.34	12	5,361	0.26	425
rs1564573	CSMD1: Intron Variant	0.42	20	1,858	0.08	27
rs2449184	CSMD1: Intron Variant	0.42	33	3,760	0.17	0
rs1803274	BCHE: Missense Variant	0.20	23	221	0.01	9
rs948028	GRIK4: Intron Variant	0.16	38	14,922	0.78	2
rs12779623	MALRD1: Missense Variant	0.20	48	303	0.01	1

For the GWAS analysis, if considering 0.05/19,276 = 2.59e-6 as the significance level for *p*-value after the Bonferroni correction, then 120 out of 930 (12.9%) HTN patients can be assigned a significant SNP identified by GWAS; even with a relaxed significance level of 1.09e-5, only 146 out of 930 (15.7%) HTN patients are covered or explained by these significant GWAS SNPs (Fig. 4A). This suggests that the significant SNPs of HTN identified at the population level by GWAS do not necessarily exist in a given HTN patient, leaving a significant portion of HTN patients unexplained by these significant SNPs.

We examined the number of minor-allele SNPs existing in each HTN patient (with the value of minor-allele SNP as '1’), and we further calculated the average number across all 930 HTN patients. On average, there are 7,767minor-allele SNPs (out of 19,276 total SNPs) existing in HTN patients. Assuming all these existing risk alleles have the same prior probability of causing HTN, and only one of them is causing HTN, then the prior probability for each risk allele is 1.0 / 7767 = 1.3e-4. Interestingly, the top one SNP selected by IBI for each HTN patient has a much higher posterior probability, ranging from 0.08 to 0.99 (Fig. 4C). If considering 0.1 as a significant posterior probability threshold, then 922 out of 930 (99.1%) HTN patients can be assigned a significant IBI SNP as the potential cause for their HTN status; with a more restrictive threshold of 0.2, 741 out of 930 (79.7%) HTN patients can be explained. These results provide support that IBI is able to find a top SNP with significant posterior probability (>=0.1) of influencing HTN, relative to random chance (1.3e-4), for the majority of HTN patients as a potential genomic cause.

As shown in Table 1, the intronic SNP rs13265032 in the *CSMD1* loci is assigned as the top-1 SNP by IBI for 46% (425) of 930 HTN patients. It was also ranked high (12) by IBI ${M}_{s,r}$ among all the SNPs. By contrast, this SNP was never assigned as a top-1 SNP for any HTN patient by GWAS and this SNP was ranked lowly (5,361) by GWAS among all the SNPs. Interestingly, other intronic SNPs in the *CSMD1* loci have been reported to be associated with hypertension [25] or blood pressure response to hydrochlorothiazide [26, 27], an antihypertensive drug. Among the 86 SNPs located in the *CSMD1* loci in our dataset, three of them were ranked highly by IBI, as shown in Table 1, while all of them were ranked relatively low by GWAS. These three novel SNPs identified by IBI in the *CSMD1* loci provide evidence to support the reported role of *CSMD1* in HTN, which may warrant further analysis for their potential causal influence on *CSMD1* regulation. In addition to SNPs in the *CSMD1* loci, IBI also identified a novel missense variant of rs1803274 in the *BCHE* loci, a novel intron variant rs948028 in the *GRIK4* loci, and a novel missense variant of rs12779623 in the *MALRD1* loci as the top-1 likely cause of HTN in 9, 2 and 1 HTN patients, respectively (Table 1). Interestingly, *BCHE* [28, 29] loci, *GRIK4* [30] loci and *MALRD1* loci [4] have been reported to be associated with blood pressure regulation, although GWAS analysis ranked their SNPs relatively low (Table 1). Overall, these results provide support for IBI being able to identify novel and biologically meaningful SNPs or genes associated with HTN that were missed by GWAS analysis.

### IBI found well-known significant variants or genes that were missed by the parallel GWAS analysis in the same cohort

We list several missense variants (Table 2) as well as the gene loci (Table 3) that were previously reported for their influence on blood pressure regulation, beyond the ones discussed in Table 1. In Tables 2 and 3, IBI ranks were determined by ${M}_{s,r}$ while GWAS ranks were determined by the *p*-value.

**Table 2:** SNPs well-known for blood pressure regulation identified by IBI but missed by GWAS

rs ID	Genes: Variant type	MAF	IBI rank	GWAS rank	*p*-value
rs11575542	DDC: Missense Variant	0.01	79	599	0.02
rs37369	AGXT2: Missense Variant	0.08	93	748	0.03
rs723580	CLIC5: Missense Variant	0.04	189	11851	0.61

**Table 3:** Genes well-known for blood pressure regulation identified by IBI but ranked relatively low by GWAS

rs ID	Genes: Variant type	MAF	IBI rank	GWAS rank	Top GWAS rank	*p*-value
rs3211938	CD36 [31, 32]: Stop Gained	0.01	32	141	141	3.8E-03
rs6730396	ALLC [33]: Missense Variant	0.01	45	192	192	5.5E-03
rs9896904	ANKFN1 [26]: Intron Variant	0.07	57	14392	2130	7.5E-01
rs11899922	THSD7B [4, 34]: Intron Variant	0.07	70	9415	929	4.8E-01
rs10968668	LINGO2 [35, 36]: Intron Variant	0.08	80	1237	1237	4.9E-02
rs13261739	PDGFRL [37, 38]: Intron Variant	0.13	94	7156	7156	3.6E-01
rs6140644	PLCB1 [39]: Intron Variant	0.17	116	9947	699	5.1E-01
rs7647302	KCNAB1 [40]: Intron Variant	0.01	158	9528	1964	4.9E-01

In Table 2, the missense variant of rs37369 [41] has been shown to be one of the four functional SNPs of AGXT2, which has been reported to have strong associations with several cardiorenal traits, such as coronary heart disease [42]. Its significant association with hypertension was recently reported via multiple regression analysis involving only several targeted SNPs [43]. The missense variant rs11575542 was recently identified as a functional variant of the DOPA Decarboxylase (*DDC*) gene during systematic polymorphism screening across the 15-Exon *DCC* locus [44]. The SNP was shown to alter the enzyme activity of DCC and result in changes in renal Dopamine excretion that is linked to hypertension [44]. The missense variant of rs723580 was reported to be a top trans-eSNP [45] for the expression level of EPO associated with the red blood cell traits that were strongly linked to hypertension [46]. With low MAF in our relatively small discovery cohort, these three SNPs were ranked much higher by IBI than by the parallel GWAS analysis (Table 2). This result provides support that IBI can recognize biologically meaningful genomic variants of low MAF, relative to GWAS, particularly when the sample size is small compared to the number of SNPs to be tested.

Table 3 shows a list of genes reported to be associated with BP regulation or HTN where at least one related paper was found for each gene’s association with BP. IBI identified these genes as candidate genes influencing HTN since these gene loci contain at least one SNP that is highly ranked by IBI for its association with HTN. Interestingly, all these genes were ranked relatively low by GWAS even considering the highest SNP rank by GWAS (‘Top GWAS rank’ in Table 3) within each gene locus; all of these novel SNPs were also ranked relatively low by GWAS (‘GWAS rank’ in Table 3) with non-significant *p*-values (Table 3). Moreover, among these eight SNPs highly-ranked by IBI and lowly-ranked by GWAS, six have MAF lower than 0.1 and three have MAF as low as 0.01 (Table 3). Tables 2 and 3 provide support that IBI is better able than GWAS to identify significant variants of low MAF.

### IBI top SNPs identify genetic risk scores that are more predictive for HTN than do the GWAS top SNPs

**Fig. 5:**
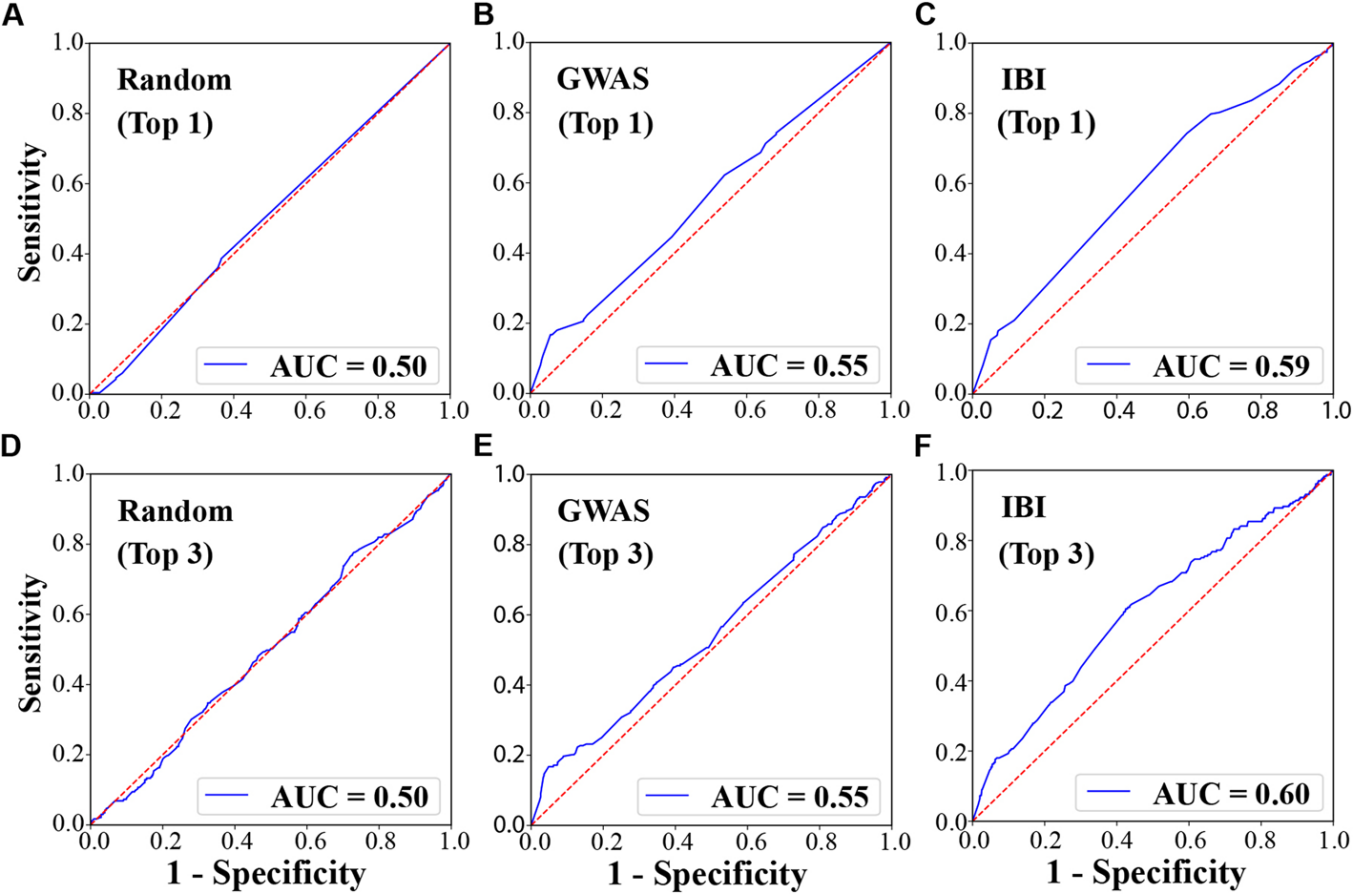
ROC curves for predicting hypertension from top SNPs. The top-1 SNP (**A**, **B**, **C**) and top 3 SNPs (**D**, **E**, **F**) selected by GWAS (*p*-value ranking), IBI (${M}_{s,r}$ ranking) or randomly were used to calculate the genetic risk scores for each test patient (*n* = 1,323), which were further used to derive the AUROC for predicting hypertension. For these two experiments, random SNPs have an AUROC of 0.50, as shown in (**A**, **D**); GWAS-selected top SNPs have AUROC of 0.55, as shown in (**B**, **E**); IBI-selected top SNPs have AUROC of 0.59 for top 1 SNP and 0.60 for top 3 SNPs, as shown in (**C**, **F**)

We further compared the capabilities of significant SNPs, identified by IBI and GWAS, in predicting HTN. After running IBI and GWAS on the training (discovery) dataset, we were able to rank all the SNPs based on ${M}_{s,r}$ derived from IBI or *p*-values obtained from GWAS. For each subject in the test set (*n* = 1,323), based on the IBI ranking or GWAS ranking, we identified the top 1 SNP and the top 3 SNPs that exist in this subject (with a value of ‘1’ denoting a minor allele). We then used these top SNPs to calculate the genetic risk scores (GRS) for each subject as the sum of their risk (minor) alleles, weighted by the odds ratio for GWAS and by ${M}_{s,r}$ for IBI. We used min-max normalization to normalize both the IBI and GWAS GRS to avoid potential bias from the different scales of the original values. We then directly calculated the area under the ROC curve (AUROC or AUC) using the normalized GRS for each patient (Fig. 5). We also trained a logistic regression model for HTN prediction using this feature of normalized GRS, which gave very similar results (data not shown).

As expected, using randomly selected SNPs showed poor prediction performance, with an AUROC of 0.50 (Fig. 5A, D); the GWAS-selected top one or three SNPs both have an AUROC of 0.55 (Fig. 5B, E) suggesting some level of prediction; the IBI-selected top SNP had an AUROC of 0.59 (Fig. 5C) and the top three SNPs had an AUROC of 0.60 (Fig. 5F). To understand whether the improvement of AUROC by the IBI-selected SNPs is statistically significant, compared to that by the GWAS-selected SNPs, we used the R package of *pROC* that is dedicated to comparing ROC curves [47]. The *p*-values for comparing the top-1 and top-3 SNPs ROC curves between GWAS and IBI are 0.05 and 0.01 specifically, suggesting a statistical significance. The statistically higher AUROC achieved by IBI provides support that the top SNPs it selects predict hypertension better than the top SNPs selected by GWAS.

## Discussion

In this study, we developed and applied a novel and individualized method (IBI) to estimate the personalized genomic variants for the complex trait of hypertension. We compared its performance with the population-based GWAS method using a real dataset from the FHS cohort. The significant overlap of the top-ranked SNPs by both IBI and GWAS suggests a degree of agreement between these two approaches. On the other hand, the unique SNPs they found support a complementary role of IBI to current GWAS analyses. Interestingly, by focusing on each individual and its patient-like-me subgroup, IBI could identify significant SNPs of low MAF in the same cohort, relative to GWAS. IBI was also able to identify more diverse and individualized top SNPs to explain the HTN patients. Moreover, the top SNPs identified by IBI from the discovery cohort were able to predict HTN better than the top ones derived from GWAS when applied to an unseen test cohort. We also identified evidence from the literature to support the biological significance of top SNPs found by IBI, especially the ones highly-ranked by IBI and lowly-ranked by GWAS. In summary, our study provides support that IBI can serve as a complementary approach in discovering novel and personalized genomic variants that may be missed by GWAS.

Contemporary GWAS studies often involve using large sample sizes (~1 million) to gain sufficient power, especially for variants of low MAF. Considering the large genomic heterogeneity among different individuals, as well as the nature of complex diseases often being affected by many variants of small effect size, an alternative approach is to focus on the subpopulation containing the specific variant of low MAF under evaluation, as IBI does. In this way, IBI may be able to better evaluate the effect of low-MAF variants in a patient-like-me subpopulation, without the potential noise from a large remaining population not containing such variants; moreover, this large remaining population could be explained better with a remaining-population driver. The fact that the top-ranked SNPs by IBI in general have a higher overall marginal likelihood, ${M}_{s,r}$, and higher information gain with respect to the HTN status, provides support that IBI may have found specific drivers that better explain the subpopulations. Our results also support that IBI is not compromised in identifying significant high-MAF SNPs. IBI’s population partition strategy aligns well with the concept of personalized medicine in which different individuals or subpopulations may have different underlying genomic influences on producing complex clinical phenotypes such as HTN.

As a general Bayesian framework, IBI can be applied to any discrete trait. It can also be applied to continuous traits by changing the marginal likelihood function from using the BDeu score for discrete variables to using the Bayesian information criterion (BIC) score for continuous variables such as blood lipid levels [14]. The traits can be clinical traits such as HTN, or molecular traits such as gene expression from microarray or RNA-seq data. The genomic data can be genotyping array data or whole genome sequencing (WGS) data; indeed, IBI has a significant potential in detecting rare or low-frequency genomic variants from the rapidly-accumulating WGS data.

For the current approach presented in this study, one limitation is that it only considers the genomic factors of HTN, while not modeling the effects of other factors such as age, sex, population structure, and the family relatedness that may exist in this FHS cohort. To model the effects from non-genomic factors, we plan to incorporate linear mixed models [48–52] into our current framework. Also, due to confounding factors such as population structure, as well as linkage disequilibrium (LD), the influencing variants described in this paper are not necessarily causal. Further fine mapping approaches, functional analysis, or Mendelian randomization can be used to further pinpoint the potential causality. Another interesting future direction is to search for more than one genomic variant that might work together to affect and influence the phenotypes of individuals and subpopulations.

## Conclusions

In summary, we described a novel Bayesian method for identifying personalized genomic variants that influence complex traits, such as HTN. IBI can serve as a complementary approach to GWAS, especially in detecting significant genomic variants of low frequency. The novel SNPs we identified for HTN warrant further analysis for their possible causal role in blood pressure regulation.

### Supplementary Information

**Additional file 1.** The top 188 SNPs ranked by *p*-value. Corresponding columns include rsID, CHR, gene, MAF, *p*-value,*M*_*s*_, GPP, *M*_*s,r*_, Rank_*p*-value, Rank_*M*_*s*_, and Rank_*M*_*s,r*_.

**Additional file 2.** The top 188 SNPs ranked by *M*_*s*_. Corresponding columns include rsID, CHR, gene, MAF, *p*-value, *M*_*s*_, GPP, *M*_*s,r*_, Rank_*p*-value, Rank_*M*_*s*_, and Rank_*M*_*s,r*_.

**Additional file 3.** The top 188 SNPs ranked by *M*_*s,r*_. Corresponding columns include rsID, Chromosome (CHR), gene, MAF, *p*-value, *M*_*s*_, GPP, *M*_*s,r*_,Rank_*p*-value, Rank_*M*_*s*_, and Rank_*M*_*s,r*_.

**Additional file 4.** The Information gain values for the top 188 SNPs ranked by IBI and GWAS respectively, as well as 188 SNPs selected at random. The method column specifies the method (such as IBI or GWAS) used to identify the SNPs.

**Additional file 5.** The 16 unique top-1 SNPs selected by GWAS. Corresponding columns include rsID, CHR, gene, MAF,*p*-value, *M*_*s*_, *M*_*s,r*_,and #HTN patients explained.

**Additional file 6.** The 25 unique top-1 SNPs selected by IBI. Corresponding columns include rsID, CHR, gene, MAF,*p*-value, M_*s*_, *M*_*s,r*_, and #HTN patients explained.

## Data Availability

The genomic and blood pressure data from the FHS cohort (study accession: phs000007.v30.p11) are publicly available via controlled-access through the database of Genotypes and Phenotypes Study (https://www.ncbi.nlm.nih.gov/projects/gap/cgi-bin/study.cgi?study_id=phs000007.v30.p11). The source code for IBI together with additional codes and summary statistics data for reproducing the figures is publicly available on GitHub at https://github.com/asadcfc/IBI.

## Abbreviations

GWAS: Genome-wide association study
IBI: Individualized Bayesian inference
TCI: Tumor-specific causal inference
HTN: Hypertension
BP: Blood pressure
SBP: Systolic blood pressure
DBP: Diastolic blood pressure
FHS: Framingham heart study
MAF: Minor allele frequency
CBN: Causal Bayesian network
BDeu: Bayesian Dirichlet equivalent uniform
TOPMed: The Trans-Omics for Precision Medicine
IG: Information gain
GRS: Genetic risk score
AUROC or AUC: Area under ROC curve
BIC: Bayesian information criterion
